# Antimicrobial s-PBC Coatings for Innovative Multifunctional Water Filters

**DOI:** 10.3390/molecules25215196

**Published:** 2020-11-08

**Authors:** Emanuele Luigi Sciuto, Simona Filice, Maria Anna Coniglio, Giuseppina Faro, Leon Gradon, Clelia Galati, Natalia Spinella, Sebania Libertino, Silvia Scalese

**Affiliations:** 1 Istituto per la Microelettronica e Microsistemi–Consiglio Nazionale delle Ricerche (CNR-IMM), Ottava Strada 5, 95121 Catania, Italy; sebania.libertino@imm.cnr.it (S.L.); silvia.scalese@imm.cnr.it (S.S.); 2 Azienda Ospedaliero Universitaria Policlinico “G. Rodolico-San Marco”, Via Sofia 78, 95123 Catania, Italy; 3 Department of Medical and Surgical Sciences and Advanced Technologies “G.F. Ingrassia”, University of Catania, Via Sofia 87, 95123 Catania, Italy; ma.coniglio@unict.it; 4 Azienda Sanitaria Provinciale di Catania, Via S. Maria La Grande 5, 95124 Catania, Italy; giuseppina.faro@aspct.it; 5 Faculty of Chemical and Process Engineering, Warsaw University of Technology, ul. Warynskiego 1, 00-645 Warsaw, Poland; leon.gradon@gmail.com; 6 ST Microelectronics, Stradale Primosole 50, 95121 Catania, Italy; clelia.galati@st.com (C.G.); natalia.spinella@st.com (N.S.)

**Keywords:** polypropylene, biofilm, s-PBC, sulfonated polymers, antibacterial coating, filter, *Pseudomonas aeruginosa*

## Abstract

Biological contamination is a typical issue in water treatment. Highly concentrated microbial suspensions in a water flow may cause filter occlusion and biofilm formation, affecting the lifespan and quality of water purification systems and increasing the risk of nosocomial infections. In order to contrast the biofilm formation, most of the conventional strategies rely on the water chemical modification and/or on the use of filters functional coatings. The former is unsafe for huge chemicals spilling required; therefore, we focus on the second approach and we propose the use of a sulfonated pentablock copolymer (s-PBC, commercially named Nexar™) as innovative multifunctional coating for improving the performance of commercial water filters. S-PBC-coated polypropylene (PP) samples were tested against the pathogen *Pseudomonas aeruginosa*. The covering of PP with s-PBC results in a more hydrophilic, acid, and negatively charged surface. These properties avoid the adhesion and proliferation attempts of planktonic bacteria, i.e., the biofilm formation. Inhibition tests were performed on the as-modified filters and an evident antibacterial activity was observed. The results point out the possibility of using Nexar^TM^ as coating layer for filters with antifouling properties and a simultaneous ability to remove bacteria and cationic dyes from water.

## 1. Introduction

Environmental pollution is one of the current global challenges requiring innovative methods and systems to preserve human health [1,2,3]. In this context, new, cheap, efficient, and safe methods to monitor and purify the natural resource of water are urgently needed. Innovative multifunctional coatings represent an emerging appealing technology aimed to improve the performance of commercial filters. Recently, advanced membranes made from new materials and approaches have been employed in water treatment [4] including microfiltration (MF), ultrafiltration (UF), reverse osmosis (RO), and nanofiltration (NF) membranes. The filtration technology is very easy and straightforward to use. Depending on the type of destination, water filtration is, generally, performed by a multi-step sample processing in which the water flow interacts with a cascade of functionalized filters in order to get a combined purification (biological, chemical, and/or mechanical). Moreover, filtration is cost effective since it does not require high maintenance costs. Furthermore, filtration does not affect the odor and taste of the water with respect to other purification technologies, such as adsorption and photocatalysis, and does not introduce nanoparticles (NP) in water, avoiding the issue of NP safe recovery [5,6]. As an alternative to their use, polymeric films could be used as filters in order to remove inorganic and organic contaminants, such as particulate and biomass from water [7,8], reducing the chlorine or other chemicals demand for disinfection and controlling the taste and odor compounds [9].

A big issue in the use of filters for water treatment is their robustness and lifespan inside the dedicated facilities. Their cleaning and care is vital to allow the water purification process for a long time and at low-pressure values, reducing both processing and maintenance costs. For example, filters have a high risk of impurities stacking inside, requiring their replacement every 6 to 12 months (i.e., the lifespan for most of the conventional depuration devices such as the sediment, activated carbon, and UV-based filters) [10]. Innovative approaches may include the possibility to actively monitor the filter status by using miniaturized sensing systems on which the research is on-going [11,12,13], but such an approach would require a strong technological effort.

One of the elements affecting the filter functioning is the biomass, mostly composed of bacteria, that can clog the filter meshes creating a biofilm on its surface. Biofilm is a complex aggregation of surface-associated microorganisms encapsulated inside a secreted extracellular polymeric substances (EPSs) matrix [14]. This assemblage is the result of an attachment, colonization, and growth process that follows the interaction of planktonic bacteria with a surface (i.e., the filter surface in a water depuration system). The electrostatic forces operating between the bacterial membrane and the target surface induce the attachment of bacteria and, then, the bacteria pili, fimbriae, and EPSs secretion make the interaction stronger and irreversible. Once aggregated, the microorganisms start colonizing the surface and growing into microcolonies encapsulating themselves inside the EPSs matrix and consolidating the complex biofilm architecture. This growth is genetically regulated by a quorum sensing process: the surface-associated bacteria exchange some autoinducer molecules with each other that interact with specific activator and suppressor sequences inside the microbial genome. At high mass level, bacteria are released and dispersed outside the mature biofilm in a detachment process induced by intrinsic and extrinsic factors, such as enzymes, able to breakdown the biofilm matrix [15]. The detachment is very important in the case of pathogens that are crucial for water safety and healthcare, such as the gram-negative *Pseudomonas aeruginosa*. It is an opportunistic pathogen affecting human airway, urinary tract, eyes, and, sometimes, blood. It is considered one of the biggest causes of nosocomial infections, affecting immunocompromised individuals, and is generally transferred by the hospital environmental sources, such as the water system. Water faucets in hospitals can become reservoirs for *P. aeruginosa*, especially in the presence of its mature biofilm [16,17]. In *P. aeruginosa* biofilms, nonmotile cells form a rigid wall surrounding a hollow structure. These structures result in the release of individual cells or eruption of cell aggregates from the interior of the cluster, contaminating the outcoming water and increasing the risk of infection [15].

The strategies adopted to limit the biofilm formation on water filters are mostly focused on the chemical composition of water inside the depuration system on the intrinsic nature of the filter surface and on its modification by functional coatings [18,19].

The water composition can be modulated by the addition of biocides, such as oxidizing disinfectants, surfactants, and antibiotics, that affect the bacteria physiology in the biofilm. However, the mature biofilm itself can act as a barrier avoiding the biocides molecules to diffuse inside it and be effective against the encapsulated microorganisms. Therefore, a large amount of chemicals is required to achieve a complete disinfection resulting in high costs and in the release of unsafe compounds [20,21,22].

The nature of the targeted surface, i.e., its topography, hydrophilicity/hydrophobicity, and electrostatic composition, is also crucial to modulate the attachment process. In particular, a smoothed, hydrophilic, and neutral surface can expose bacteria to the shear stress caused by the water flow hydrodynamics and limit the electrostatic interactions in the adhesion process, thus avoiding the attachment and colonization during the biofilm formation. However, the intrinsic nature of filter surface cannot sufficiently oppose to the microbial adhesion in case of high-density biomass in the water flow [23,24,25].

Deep-bed filters used for water cleaning are made of polypropylene (hydrophobic material) fibers with an average fiber diameter of 5 micrometers and a porosity of order 90%. The structure of the filter depends on the particular operational condition, i.e., flow rate of water and concentration of contaminant. Microorganisms, due to their geometry and size, easily deposit on the fiber surface gradually filing a filter space. In addition, the void space of the filter is reduced due to bacteria colonies growth. The biofouling effect, which results from such dynamics, causes a growth of local shear-stress and possible re-entrainment of collected deposits. Reduction of the biofouling significantly improves filter performance with reduction of pressure drop of the flow and increases life-time of the filter use.

A different approach to guarantee the filter preservation is represented by the use of surface functional coatings. The antimicrobial coatings concern the chemical modifications of a surface in order to contrast the biofilm formation at various levels: part of them are based on an anti-adhesive principle, i.e., preventing bacteria to adhere; others adopt bactericidal strategies, i.e., killing the microorganisms either before or after contact with the surface [26,27]. The anti-adhesive coatings enrich the surface with a steric, mechanic, and/or electrostatic barrier that block bacteria in the first attachment. Some of them, for example, can be obtained by the polymerization of negatively charged molecules increasing the electrostatic repulsion and keeping away the microorganisms from the filter surface [28]. An interesting anti-adhesive coating is the one based on the “polymer brushes” activity obtained through a binding process of hydrophilic polymers. Microorganisms that are basically hydrophobic are sterically repelled when they interact with these hydrophilic brushes [29,30].

The biocidal coatings are chemical modifications of a surface integrating different biocidal agents. They contrast the biofilm formation interfering with the physiology of both planktonic and attached bacteria at different levels, thus causing their massive death [31,32]. Examples of biocidal coatings are metal-based derivatization, such as the silver polymeric nanocomposites functionalization [33], and copper and zinc oxides dosing into water-soluble or insoluble matrices [34].

Recently, carbon nanomaterials such as graphene oxide and reduced or modified graphene oxide have been tested as cheap and safe alternative to chlorine compounds [35] opening the possibility of testing these materials also as antimicrobial coating taking advantage of their hydrophilicity and sheet like morphology.

The risk of disinfection byproducts in water due to the use of chemicals may be overpassed by the “contact killing” biocidal coatings. These surfaces incorporate and expose biocides blocking the adhesion and growth processes of interacting bacteria without reagents spilling and dispersion in water. Some contact killing coatings have been developed using the antimicrobial peptides. These peptides are natural biocides causing the microbial death through their membrane disruption and pore formation, and/or the inhibition of proteins, cell wall, and enzymes synthesis [36,37].

Considering all the advantages and drawbacks of both anti-adhesive and biocidal coatings, the best solution in terms of surface coatings and filter structure preservation could be found in implemented multifunctional approaches incorporating both the repulsion and contact killing mechanisms. Taking advantage of its hydrophilicity, acidity, and negative surface charge, we present here for the first time the use of Nexar^TM^, a pentablock-sulfonated copolymer (s-PBC: tert-butyl styrene, hydrogenated isoprene, sulfonated styrene, hydrogenated isoprene, tert-butyl styrene (tBS-HI-SS-HI-tBS)) as a coating for commercial filters, such as a polypropylene (PP) ones, in order to prevent biofilm formation on this surface. The molecularly designed Nexar™ structure results in a polymer with controlled swelling and good mechanical properties in the hydrated state within the advantages of low cost, good processability, and ease of functionalization. In addition, this type of precursor has a micellar structure [38] that can be modulated at the polymeric level in order to form preferential channels for filtration application such as in desalination [39] or for application as a proton exchange membrane in water electrolysis [40]. In particular, for water treatment applications, s-PBC itself has been already used as adsorbent for heavy metals removal [41] or in combination with known photocatalysts for azo-dye degradation [42,43]. In addition, it was recently found to have also remarkable antibacterial properties [44]. We investigated the possibility to use Nexar™ as multifunctional coating to modify a polypropylene (PP) surface. The anti-adhesive and bactericidal activity of modified PP filter with respect to bare ones were investigated by a biofilm induction experiment and an antimicrobial susceptibility (Zone of Inhibition) test of *P. aeruginosa*. The obtained results confirm the advantageous use of Nexar™ as antifouling and antibacterial coating layer for commercial filters in water purification application.

## 2. Results

### 2.1. Biofilm Adhesion and Surface Properties

**Figure 1:**
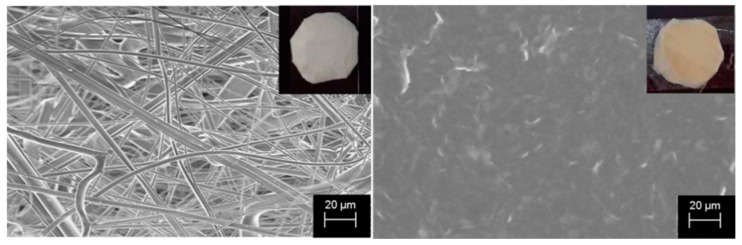
Photos and SEM images of polypropylene (PP) (on the left) and sulfonated pentablock copolymer (s-PBC)@PP (on the right) coupons.

The morphology of untreated and covered PP coupons was investigated by scanning electron microscopy (SEM) analysis and images are reported in Figure 1. The insets show the two samples photos.

The initial filter (on the left in the figure) is white and is formed by randomly distributed fibers with diameters of around 5 microns. After s-PBC deposition, the coupon surface turns to yellow and the s-PBC completely covers the PP fibers as shown in the image on the right, resulting in a homogeneous and smooth surface. As already mentioned, smooth surfaces may reduce the bacteria chance of attachment on the filter surface.

**Figure 2:**
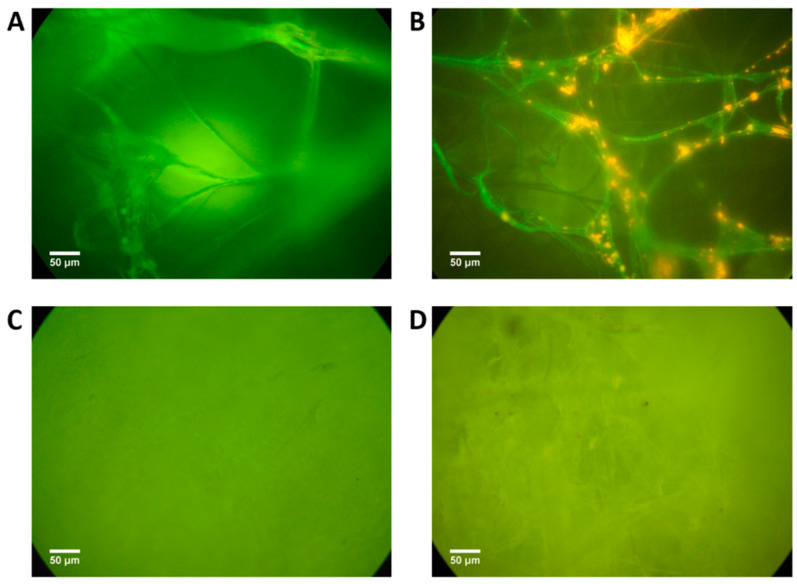
Fluorescence microscopy images of reference PP and s-PBC@PP coupon before (**A**,**C**, respectively) and after (**B**,**D**, respectively) 20 days of incubation with *Pseudomonas* *aeruginosa* in water.

The antimicrobial power of the s-PBC polymer was studied starting from its ability to avoid the bacterial attachment and the biofilm engraftment. To this purpose, we analyzed the biofilm formation of *P. aeruginosa* on s-PBC@PP and reference coupons after incubation in water. Figure 2 reports the fluorescence optical image of PP and s-PBC-covered PP filters before (A and C, respectively) and after (B and D, respectively) 20 days of incubation with *P. aeruginosa* in water.

The optical images of starting coupons (Figure 2A,C) confirm the morphology observed by SEM analysis: PP coupon is formed by microfibers that are completely covered by s-PBC deposition resulting in a more homogeneous and smoother surface.

Once exposed for 20 days to the microbial suspensions, bacterial aggregates of *P. aeruginosa* appeared on the PP coupon (Figure 2B) as orange-stained spots (typical staining for dead bacteria, see Materials and Methods section), contrasting the green intrinsic fluorescence of polypropylene fibers. In this case, cells managed to stick to the PP surface and started to aggregate. However, probably the lack of nutrients affected the *P. aeruginosa* physiology and prevented the colonization of the rest of fibers and the biofilm formation. On the other hand, no traces of bacteria (no aggregates) are visible on the exposed s-PBC@PP coupon (Figure 2D) that maintained the same starting appearance. After a prolonged incubation in water, the modified coupons remained completely free from any bacterial aggregates, suggesting that the treated surface blocked any attachment attempts of the suspended cells. The ability of the s-PBC layer to avoid bacteria attachment and the growth of biofilm can be ascribed to the following reasons: (1) a more homogenous and smoother surface of the modified coupon with respect to the initial PP filter; (2) the hydrophilic character of s-PBC; (3) the negative charge of sulfonilic groups of the s-PBC layer (see Figure S2 of SI). As discussed in the introduction, all these properties are fundamental for anti-biofouling coatings.

Previous studies [40,41] performed by FT-IR analysis on s-PBC structure demonstrate that its molecular structure in polar solvent does not undergo chemical modification with respect to the commercial material. In fact, the IR absorption peaks characteristic of SO_3_H groups were unmodified after dispersion in polar solvent. These are responsible of the negative charge character of s-PBC coating, as also confirmed by its ability to adsorb positively charged molecules [42,43]. Furthermore, the presence of IR absorption peaks due to the presence of hydroxyl groups confirmed the high hydrophilic character of s-PBC both in the dry and wet state. This characteristic is transferred to the surface of PP coupons after s-PBC deposition, as shown by water uptake measurements and surface hydrophilicity. The change from hydrophobic to hydrophilic behavior of the PP surface after the deposition of s-PBC was confirmed by contact angle measurements (see Figure S3 of SI). The initial PP surface is totally hydrophobic. On the contrary, the PP surface covered by the s-PBC layer is hydrophilic. This is in agreement with water uptake values showing that the s-PBC layer increases the water uptake of PP by a factor of about 50. These values were measured for three PP and three s-PBC@PP coupons, and the average value obtained is 3% for PP and 160% for s-PBC.

In order to further point out the modification induced by s-PBC covering on the PP surface, the dyes adsorption properties of untreated and modified filters were investigated. Both PP and s-PBC@PP filters were tested for the adsorption of cationic and anionic dyes, i.e., methyl orange (MO) and methylene blue (MB), respectively. PP filters used as references were not able to adsorb MO nor MB, independently on the dye superficial charge because of their hydrophobicity. S-PBC confers to PP filter a negative and acidic surface, and thus the s-PBC@PP is not able to adsorb anionic molecules, as in the case of MO.

**Figure 3:**
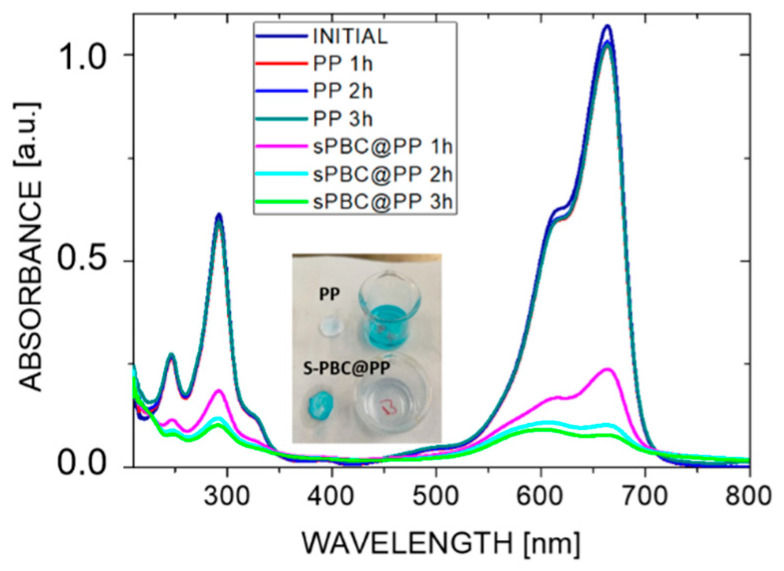
UV–Visible absorbance spectra of methylene blue (MB) solutions after dipping PP and s-PBC@PP for 1, 2, or 3 h. In the inset, a photo of the filters and dyes solutions after three hours of adsorption.

Figure 3 reports the UV–Visible absorbance spectra of MB solutions where PP and s-PBC@PP filters were dipped in dark for three hours. Spectra for MO solutions are not reported here since both the initial PP filters and covered ones were not able to adsorb it.

Methylene blue is a cationic, thiazine dye, which absorbs light at 664 nm (n-∂*) (monomer) with a shoulder at 610 nm corresponding to the dimer. In concentrated aqueous solutions, aggregation occurs and MB aggregates are easily detectable by UV–Vis spectroscopy since these absorb light at lower wavelengths with respect to the monomer [42]. The MB adsorption efficiency confirms the negative and acid character of the s-PBC surface. The MB absorbance peak in solution decreased by increasing the contact time with s-PBC@PP: the main adsorption occurred in the first hour of contact, the process continues during the second hour, in fact, the peak further decreases, and it stops, providing a constant peak in solution, after the last one. After the second hour, the absorbance peak slightly shifts to lower wavelengths and the relative ratio of the peak at 664 nm vs. 610 nm is reduced underlining the formation of MB higher aggregates. The quantitative analyses, % of MB removal, and amount of adsorbed MB molecules as millimoles (mmol) per mg of s-PBC@PP filter are summarized in Table 1. The same parameters are reported for PP filters used as reference.

The PP filter absorbs ~5% of MB, thus removed from the solution, after the first hour and then the process stops. By covering this surface with s-PBC, a strong increase of MB removal efficiency is observed, up to 92% after three hours, thanks to the hydrophilic, acid, and negative character of this surface. The amount of adsorbed MB molecules as mmoles/mg of coupon increased of one order of magnitude after s-PBC coverage, i.e., 0.15 mmoles/mg. This result is also clearly visible by observing the color of coupons and MB solutions after three hours (see inset of Figure 3). The reference filter remained unaltered after being in contact with MB solution, while the modified coupon changed its color to blue and the solution became transparent after 3 h as a result of MB adsorption.

### 2.2. Bacterial Viability

**Figure 4:**
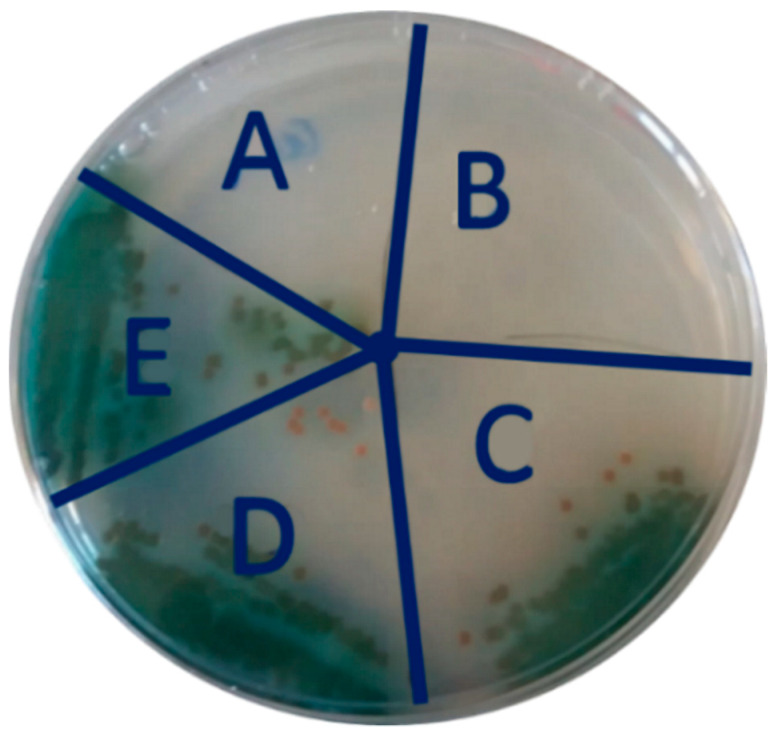
Growth test of *P. aeruginosa* at 20 days of incubation with s-PBC@PP (**A**,**B**), PP (**C**), Teflon (**D**), and glass (**E**) samples.

We have shown the anti-adhesive behavior of s-PBC coating, which allows to avoid bacteria attachment and biofilm growth. This behavior was explained because of the more acid, hydrophilic, and negative character of coupon surface after being covered by s-PBC. The second step of the investigation was focused on the survival of *P. aeruginosa* after its exposure to PP and s-PBC@PP surfaces. The bacterial suspensions used in the biofilm formation experiment (see previous sections) with PP and s-PBC@PP coupons and/or incubated with floating Teflon and glass samples (both used as references) were plated to check the microbial viability, and Figure 4 shows the results. In particular, two s-PBC@PP samples were used as replica.

*P. aeruginosa* survived after the prolonged exposure to PP, Teflon, and glass surfaces (Figure 4C–E, respectively), but not after the contact with the PP surfaces treated with s-PBC (Figure 4A,B).

**Figure 5:**
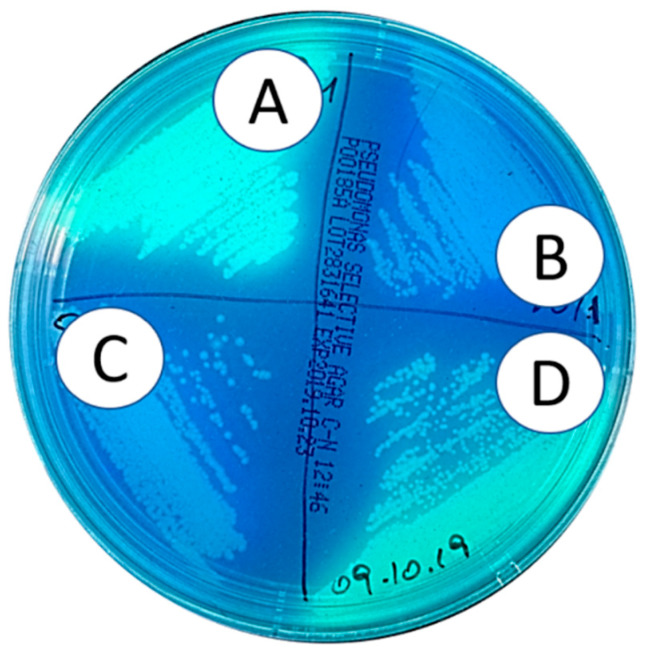
Fluorescence image of colonies of 5 × 10^6^ cells/mL (**A**,**D**) and 2.5 × 10^6^ cells/mL (**B**,**C**) *P. aeruginosa*, plated after 20 days of incubation in coupon-free water.

In order to prove that these evidences were specifically due to the coating itself, we investigated the cell viability without the floating coupons using the same minimal conditions as for the biofilm assay. Figure 5 reports the plate observed under UV light in order to excite the intrinsic fluorescence of *P. aeruginosa*. Two different *P. aeruginosa* concentrations were observed, i.e., at 5 × 10^6^ cells/mL (A and D) and 2.5 × 10^6^ cells/mL (B and C) (corresponding to the 100% and the 50% of the concentration used for the biofilm assay, respectively).

*P. aeruginosa* colonies are observed after 20 days of incubation in minimal conditions; both the number of colonies and the green fluorescence signal demonstrate that the cell physiology was not compromised by the growth minimal conditions. Moreover, the fluorescence intensity perfectly reproduced the cell concentrations, passing from the high signal of the 5 × 10^6^ cells/mL samples (Figure 5A–D) to the half signal of 2.5 × 10^6^ cells/mL samples (Figure 5B,C). This evidence confirms that *P. aeruginosa* was able to survive a long period in water and that its death is specifically related to the effect of s-PBC coating on PP support.

### 2.3. Bactericidal Effect

**Figure 6:**
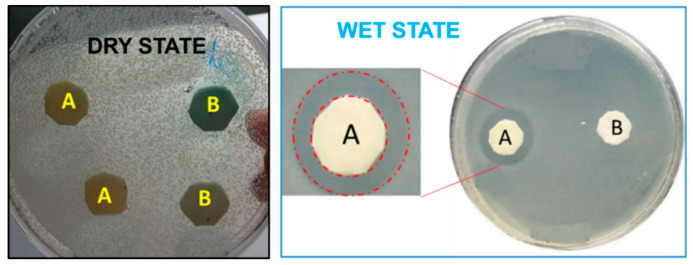
Modified Zone of Inhibition Test of *P. aeruginosa* after 24 h incubation with s-PBC@PP (**A**) and reference PP (**B**) coupons in dry state (on the left) and after adding 0.2 mL of water (on the right). The clear zone around the s-PBC@PP coupon is shown in detail (red dashed line).

The bactericidal activity of s-PBC coating was deeper investigated by a modified Zone of Inhibition Test, as described in Materials and Methods, focusing on the role of water in the observed bacterial anti-proliferative effect of polymer. The tests were performed for s-PBC@PP and PP, with the last one used as reference. The result of growth is reported in Figure 6.

**Figure 7:**
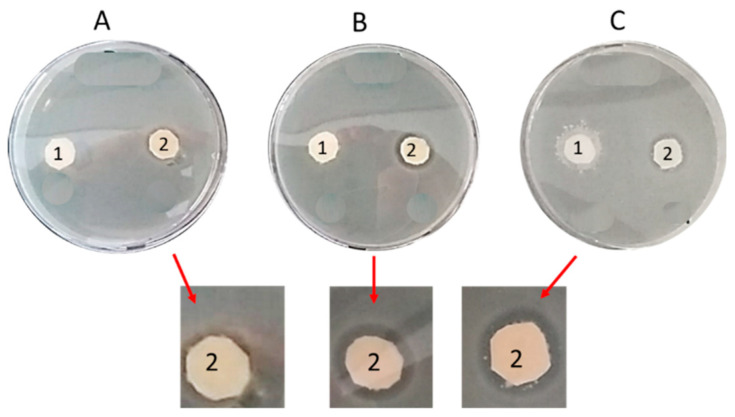
Modified Zone of Inhibition Test of *P. aeruginosa* after 24 h incubation with reference PP (1) and s-PBC@PP (2) coupons containing a residual volume of water (**A**) and after the spotting of 0.2 mL (**B**) and 0.5 mL (**C**).

In a first test, dry s-PBC@PP and reference coupons were directly faced on top of a *P. aeruginosa* plate and incubated for 24 h (Figure 6 on the left). Results show that within these drying conditions, there is no evidence of clear zones and inhibition effect for both s-PBC@PP and PP. Thus, we tested the role of water by adding it (0.2 mL) on coupons and allowing its diffusion from the material to the plate during the 24 h incubation. The result of *P. aeruginosa* growth with wet coupons is shown in Figure 6 (on the right). A clear zone (the region between the red dashed circumferences) appearing all around the s-PBC@PP coupon indicates an inhibition effect of polymer towards the *P. aeruginosa* proliferation. In the rest of the plate surface, including the surrounding area of the reference coupon (named B in Figure 6 on the right), an evident bacteria confluence proved their physiological division. These data were confirmed in triplicate (data not shown here). The experiment confirmed that s-PBC coating has a bactericidal effect only in the hydrated state. The size of the clear zone was not influenced significantly by the state of coupons solvation, as proved by the inhibition tests for PP and s-PBC@PP in different hydration states reported in Figure 7.

Changing the water volume up to 1 mL added to the coupons, the diameter of the clear zone surrounding the s-PBC@PP coupons increased moderately. Damp coupons exhibit a barely visible clear zone (A), as residual from the washing step. Adding 0.2 mL of water (B), the clear zone is well visible and no further changes were observed by increasing additional water volume from 0.2 to 0.5 (C) and 1 mL (not shown). The data suggest that the observed effect is due to a local interaction at the interface between bacteria and wet s-PBC@PP since it does not spread out at larger distances through the water medium in the plate.

Besides a high hydrophilicity, the sulfonation of a hydrocarbon chain in the s-PBC structure confers it a high degree of acidity [40,41,42,43]. As a consequence, when the polymer is in contact with water, the pH could be reduced until values that are detrimental for bacteria survival. Ref. [44] reports this theory to explain the antibacterial activity shown by Nexar^TM^ in water. To confirm this hypothesis in our experiments, we measured the change of pH for both Milli Q and sterile tap water in contact with PP and s-PBC@PP, at a volume of 0.5 mL, the one used for inhibition tests. pH values decreased after immersion of s-PBC@PP coupons in both Milli Q and sterile tap water, while no effect was observed during the immersion of PP. In particular, Milli Q water has an initial pH of 6.05 and this is reduced down to 2.5 after one hour of contact with s-PBC@PP, while sterile tap water pH reduces from 8.7 to 3.05 under the same conditions.

For inhibition tests, the observed halo could be ascribed to the acidification of small volume of water in contact with s-PBC@PP. The diameter of halo remained the same also for increasing volumes of up to 1 mL, and this underlines that the effect of this acidic surface is acted at short distance.

To investigate further, we performed inhibition tests with s-PBC@PP coupons being previously washed until their neutralization (i.e., no release of protons in Milli Q or tap water). To test the coating neutralization, we dropped on s-PBC@PP surface, before and after neutralization, a small amount of MO. This dye is usually used as pH indicator being red at acid pH and orange in neutral condition [7]. Photos of coupons with MO spotted on them are reported in Figure S4 of SI: MO turned to red after contact with s-PBC@PP coupon because of its acid surface, while MO remained orange after being dropped on the neutralized coupon, confirming the neutralization.

**Figure 8:**
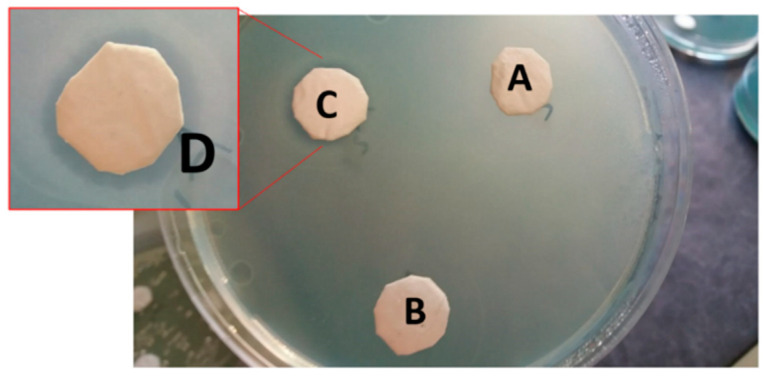
Modified Zone of Inhibition Test of *P. aeruginosa* after 24 h incubation with neutralized s-PBC@PP (**A**), PP (**B**), and standard s-PBC@PP (**C**) coupons spotted with 0.2 mL of sterile tap water. The clear zone around the standard s-PBC@PP coupon is shown in detail (**D**).

Figure 8 shows the modified Zone of Inhibition Test performed using the neutralized s-PBC@PP coupons (A) prepared as described before (see Materials and Methods). As reported in the figure, no clear zone appeared around the neutralized s-PBC@PP coupon, as for the reference PP coupon (B), while standard s-PBC@PP coupon (C) confirmed the clear zone formation. This led us to infer that the bactericidal power of the polymer depends on a charge/acid superficial effect that, if removed by pH neutralization, allows bacteria to survive and proliferate with their typical colonies spreading around the coupon.

We investigated the effect of water acidification by s-PBC by increasing the water volume: the pH strongly decreases in 0.5 mL water volumes, but remains almost constant for larger volumes up to 20 mL. In particular, 20 mL is the volume used in our biofilm growth experiment reported above and, since in this case pH remained constant, the observed death of bacteria in water could be ascribed mainly to a direct interaction of bacteria with the s-PBC@PP surface.

In general, the antimicrobial activity of s-PBC coating can be ascribed to a water acidification (for small volumes) and/or to a direct interaction (for large water volumes) when bacteria get closer to its acid surface. It is known that extreme pH affects the structure of all macromolecules, influencing most of bacteria cell component and processes such as metabolic regulation and macromolecule repair [45]. Moderate changes in pH modify the ionization of amino-acid functional groups and disrupt hydrogen bonding, inducing changes in the folding of the molecule, promoting denaturation and destroying activity. Thus, a change in H^+^ gradient inside/outside the cell can affect their survival.

**Figure 9:**
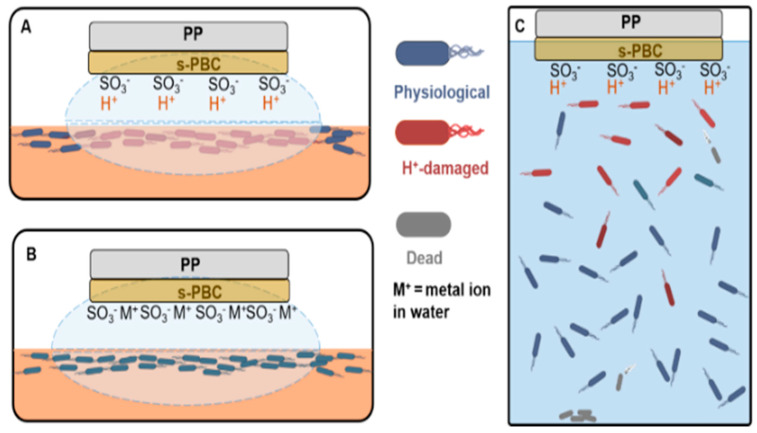
Schematic representation of *P. aeruginosa* death induced by s-PBC@PP and water: (**A**) acid s-PBC@PP in small volume system; (**B**) neutralized s-PBC@PP in small volume system; (**C**) acid s-PBC@PP in large volume system. Physiological (blue), damaged (red), and dead (gray) bacteria are indicated with different colors.

This mechanism (summarized in Figure 9) could explain the bacteria death in our experiments. In small water volume systems, used for the Zone of Inhibition Test (see before), the whole water volume (0.2–0.5 mL) is acidified, hence plated *P. aeruginosa* replication is inhibited within the water drop (red colored bacteria in Figure 9A) producing the clear zones. On the contrary, for the same water volume and in presence of neutralized s-PBC@PP (Figure 9B), water pH does not change and the bacteria survive and regularly proliferate (blue colored bacteria in Figure 9).

In large water volume systems (Figure 9C), as those used for the biofilm formation assay (20 mL), no water pH change is measured. In this case, the mobility of physiological *P. aeruginosa* (blue) in water is sufficient for them to approach the acid surface of s-PBC@PP: in proximity of this surface, the interaction of bacteria with released H^+^ species is responsible of cell damage (red) and death (gray).

## 3. Materials and Methods

### 3.1. Chemicals

Tryptic Soy Broth (TSB) was purchased from Sigma (St. Louis, MO, USA). The *Pseudomonas aeruginosa* (*P. aeruginosa*) samples were prepared using strain ATCC 15,692. Plastic Petri dishes (Ø 18 cm) were from Aptaca s.r.l. Glass flasks of 200 mL were from Simax. Multilayer polypropylene (PP) filters were in house produced using the melt-blown technology process as reported in [46] and described in the Supporting Information within a scheme of the process (see Figure S1). A sulfonated pentablock copolymer poly(tBS–HI–sS:S–HI–tBS) solution, or s-PBC, with 10–12 wt% polymer in a cyclohexane/heptane mixed solvent was provided by courtesy of Kraton Polymers LLC (Houston, TX, USA). A scheme of this copolymer, commercially available as Nexar^TM^, is reported in Figure S2 from refs. [40,41,42,43]. Ion exchange capacity (IEC) value of the commercial polymer is 2.0 meq/g corresponding to a sulfonation degree of 52 mol%. The molecular weight is 112,500 g mol^−1^ and the volume fraction (tBS–[sS:S]–HI) is 0.300–[0.226:0.208]–0.266 [38]. Taking into consideration the micellar morphology of Nexar™ [38] depending on solvent polarity and determining the morphology of the cast film, we prepared an s-PBC solution (4%wt) by dispersing the commercial s-PBC solution in a polar solvent, i.e., isopropyl alcohol (IPA).

### 3.2. Coupons Preparation

Polypropylene filters were cut into circular coupons of ~1.27 cm diameter and ~0.3 cm thickness. A 0.200 mL volume of s-PBC solution was spotted on PP coupon previously sterilized by autoclaving at standard conditions (121 °C for 30 min) in order to obtain a homogeneous coverage of its surface. The modified filter here is named s-PBC@PP. After 24 h air-drying, the deposited coupon was washed by immersion in 70% (*v*/*v*) ethanol for 10 min followed by rinsing in sterile distilled water. Each coupon was air-dried for 24 h before weighting. The amount of deposited s-PBC was ≈ 6 mg for each coupon. A PP coupon used as reference followed the same treatment procedure excluding the s-PBC deposition step.

### 3.3. Coupons Characterization

The samples morphology was characterized by a field emission scanning electron microscope (Zeiss Supra35 FE-SEM, Oberkochen, Germany) using a primary electron beam energy of 1 keV in order to avoid sample charging.

Hydrophilicity of coupons surface before and after modification was investigated by contact angle measurements using an Optical compact angle meter (CAM 200 model by KSV Instruments LTD, Helsinki, Finland). Moreover, the water uptake value of PP and s-PBC@PP, respectively, was calculated according to the following equation, by using a microbalance [40,41,42,43]:

Uptake% = [(*m*_wet_ − *m*_dry_)/*m*_dry_] × 100

where *m*_dry_ is the mass of the coupon air-dried at least for 24 h and then put into a desiccator; *m*_wet_ is the weight of the coupon after soaking it in distilled water at room temperature for 48 h and quickly wiped with a paper tissue in order to remove most of the free surface water.

In order to assay the effect of s-PBC coating on pH of water medium, PP and s-PBC@PP coupons were kept floating, with their active surface down, on 0.2; 0.5; 1; 2; and 20 mL of Milli Q and sterile tap water, respectively, for 60 min at room temperature. The initial pH of Milli Q and sterile tap water was 6.05 and 8.7, respectively. Then, the pH was measured by a micro pH-meter (Crison GLP22) and compared to the starting value. The effect of pH modulation on s-PBC antimicrobial properties was investigated by neutralizing the s-PBC@PP coupons by keeping them floating on a 0.5 mL volume of sterile tap water for about 10 min. This procedure was repeated until the water pH was stable at an initial value (5 times). Once neutralized, the s-PBC@PP coupons were analyzed by a modified Zone of Inhibition Test (see Section 3.7) by spotting 0.2 mL of sterile tap water. The neutralization and modified Zone of Inhibition Tests were performed in triplicate (replicas not shown).

Modified and unmodified coupons were tested for the adsorption of anionic and cationic dyes from water in order to assay their surface charge. For this purpose, s-PBC@PP filters were immersed in 3 mL of methyl orange (MO) or methylene blue (MB) aqueous solutions, respectively. The solutions were analyzed by recording the absorbance spectra variations of MO and MB using an UV/Vis AGILENT Cary 50 spectrophotometer in a wavelength range between 200 and 800 nm. The dyes adsorption was evaluated by the Lambert–Beer law via the absorbance peak at 465 nm and 664 nm for MO and MB, respectively. MO and MB adsorption on PP filters used as references were also evaluated in order to point out the role acted by s-PBC covering.

### 3.4. Cells Preparation

A pure culture of the reference strain *P. aeruginosa* 15,692 was prepared incubating the bacteria strain in 10 mL of TSB at 30 ± 2 °C and 150 rpm for 24 h. Subsequently, cells were harvested by centrifugation (10 min, 3600× *g*) and the pellet was washed twice in 20 mL of filter-sterilized water and suspended in 200 mL of sterile water. This suspension was incubated at 30 ± 2 °C and 150 rpm overnight, until the culture turbidity (optical density at 450 nm) was equal to 0.02, corresponding to a concentration of ≈5 × 10^7^ cells/mL. The *P. aeruginosa* suspension was then diluted 1:10 in 200 mL of sterile tap water, reaching a final concentration of ≈5 × 10^6^ cells/mL. Finally, bacterial suspension aliquots of 20 mL were spotted in the Petri dishes and used for the experiment.

### 3.5. Antimicrobial Properties of s-PBC: Biofilm Adhesion Analysis

The analysis of *P. aeruginosa* biofilm engraftment on s-PBC was performed by the following procedure: s-PBC@PP and the reference PP were kept floating, with their active surface down, on 20 mL of the *P. aeruginosa* suspension (see the Cells Preparation section) in dark for 20 days at 25 °C. Subsequently, coupons were collected and stained by spotting 100 µL of the LIVE/DEAD BacLight solution (kit from Molecular Probes Inc., Eugene, OR, USA) on their active surface, and then incubated in dark for 3 min at 22 °C. Finally, the stained coupons were fixed on the microscope slides and observed in fluorescence microscopy (using a 488 nm light of a Zeiss Axioskop microscope, Oberkochen, Germany) to check and analyze any P. aeruginosa biofilm formation. LIVE/DEAD solution is a mix of SYTO 9, which penetrates the healthy bacterial cells giving a green fluorescent signal, and propidium iodide, which diffuses only inside bacteria with damaged membranes giving a red-orange fluorescence. In parallel, a reference coupon was incubated in dark for 20 days at 25 °C floating on bacteria free water as negative reference for biofilm formation. The microscope slides (14.7 mm × 45 mm) were from Carlo Erba (Milano, Italy).

### 3.6. Antimicrobial Properties of s-PBC: Bacterial Viability Test

The *P. aeruginosa* viability was tested by sampling the water suspension after 20 days of incubation with floating coupons (s-PBC@PP and reference), performed for the biofilm engraftment analysis experiment. Teflon and glass disks, sterilized and washed using the same procedure already described for the coupons (see Section 3.2), were used as comparison, as positive and negative samples, respectively. The sample of *P. aeruginosa* suspension was picked up using an inoculation loop and plated on agar nutrient medium; the plate was then incubated at 37 °C for 24 h and checked to see the colonies formation.

The same sampling and plating procedures were adopted for a parallel analysis of 4 samples of *P. aeruginosa* (two replicas at 5 × 10^6^ cells/mL and two at 2.5 × 10^6^ cells/mL) incubated in water without coupons in dark at 25 °C for 20 days. Samples were collected after 20 days of incubation and the colonies were observed under UV light using an UVP TW-43 Transilluminator (Analytik Jena AG, Jena, Germany).

### 3.7. Antimicrobial Properties of s-PBC: Bactericidal Effect Analysis

The bactericidal effect of s-PBC was characterized by a modified Zone of Inhibition (or Kirby-Bauer) Test [47], as follows: 10 mL of 10^6^ cells/mL *P. aeruginosa* culture were spread over sterile agar nutrient plates. They were left air-drying for 30 min. s-PBC-covered coupons and references were applied on the plates keeping the active surface down; 0.2 mL and 0.5 mL of sterile tap water were spotted on top of the plate medium. Then, coupons were put on drops and plates and incubated at 37 °C for 24 h. The day after, the plates were checked to see the formation of clear zones (the zones of inhibition) around the coupons due to the bacterial growth inhibiting effect of the deposited polymer. As comparison, the same experiment was performed using damp coupons, i.e., those containing a water residual from the washing step (see Section 3.2), without spotting.

## 4. Conclusions

We proposed for the first time the application of commercial Nexar™ polymer as multifunctional coating for water filters. Our investigations proved that, once deposited on a PP filter, it is able to contrast the adhesion and proliferation of planktonic *P. aeruginosa* by a combined repulsion and contact killing mechanisms avoiding the biofilm formation. The modified surface is smoother and more hydrophilic then PP, as confirmed by the structural characterization (SEM) and contact angle and water uptake measurements. We demonstrated that the presence of sulfonilic groups in s-PBC confers it a negative surface charge and acid character, allowing absorption of positively charged dyes and repulse negative ones. All these induced characteristics make this polymer an antimicrobial coating material. It avoids bacteria attachment, growth, and survival. Its antibacterial activity relies on the acidity of this surface as shown by comparing the activity of acid and neutralized coupons. The polymer acidifies the water close to its surface causing the *P. aeruginosa* replication inhibition. It was evidenced in both Zone of Inhibition Tests and bacterial viability experiments.

This polyvalent antimicrobial nature of Nexar™ could give a substantial improvement to the surface coatings for multi-purpose filters with antifouling properties and simultaneous removal of bacteria and cationic dyes from water. Furthermore, they could allow excluding biocides spilling, increasing the filters lifespan and minimizing the biomass adhesion, leading to more efficient water purification and recycling treatments.

## Figures and Tables

**Table 1:** MB removal (%) and amount of adsorbed dye Q_t_ (mmol per mg of coupons), considering the peak at 664 nm.

	MB Removal (%)			Q_t_ (mmol/mg)		
**Time (h)**	**1**	**2**	**3**	**1**	**2**	**3**
**PP**	5	5	5	0.01	0.01	0.01
**s-PBC@PP**	77	90	92	0.13	0.15	0.15

## Supplementary Materials

The following are available online, Figure S1. Melt-blown technique for production fibrous deep-bed filters; Figure S2. Molecular structure of sulfonated pentablock copolymer (s-PBC) (Nexar^TM^); Figure S3. Optical images of contact angle measurements for PP (a,b) and s-PBC@PP (c,d) filters; Figure S4. Photos of neutralized (on the left) and acid (on the right) s-PBC@PP coupons before (on the top) and after (on the bottom) being in contact with Methyl Orange.
