# Supplementary material for: Antimicrobial s-PBC Coatings for Innovative Multifunctional Water Filters
Source: Molecules. 2020 Nov 8;25(21):5196. doi: 10.3390/molecules25215196 (PMC7664665; doi:10.3390/molecules25215196)
Supplement: Supplementary file 1 [file molecules-25-05196-s001.pdf]

Supplementary materials

Antimicrobial s-PBC coatings for innovative multifunctional water filters.

Emanuele Luigi Sciuto[a,1,§], Simona Filice[a,§], Maria Anna Coniglio[b], Giuseppina Faro[c], Leon Gradon[d], Clelia Galati[e], Natalia Spinella[e], Sebania Libertino[a] and Silvia Scalese[a]

[a] Istituto per la Microelettronica e Microsistemi – Consiglio Nazionale delle Ricerche (CNR-IMM), Ottava Strada 5, 95121 Catania, Italy;

[b] Department of Medical and Surgical Sciences and Advanced Technologies "G.F. Ingrassia", University of Catania, Via Sofia 87, 95123 Catania, Italy;

[c] Azienda Sanitaria Provinciale di Catania, Via S. Maria La Grande 5, 95124 Catania, Italy.

[d] Faculty of Chemical and Process Engineering, Warsaw University of Technology, ul. Warynskiego 1, 00-645 Warsaw, Poland

[e] ST Microelectronics, Stradale Primosole 50, 95121 Catania, Italy

*Corresponding authors: e.l.sciuto@gmail.com; simona.filice@imm.cnr.it

Preparation of PP fibrous filters

One of the most effective methods of production of fibrous depth filter is the melt-blown technique [Gradon et al., 2005]. The principle of production is shown in Fig. S1a. The granulated polymer placed in container (1) is transported with extrusion screw (2) and heated by an electric heater (3). Melted and homogenized polymer is pressed into the die (5). The rate of polymer flow is controlled by the gear system (4). The die is heated with hot air supplied from a compressor and air heater (6). The melted polymer is extruded through a row of nozzles of the die. Air nozzles surround the polymer nozzle and hot air flows along the polymer filaments. The tangential stress on the surface of the polymer filaments extend it causing reduction of the filament diameter to desired value. After solidification fibers are collected on the pivot (7), which rotates and moves back-and-forth to form a proper cartridge filter, Fig. S1b. Playing with process parameters a desired structure of the filter is obtained. The above method was used for production of fibrous layers for purpose of the paper and, in particular, the coupons used in the paper are cut out from such layers.

[1] Present address: Azienda Ospedaliero Universitaria Policlinico "G. Rodolico - San Marco", Via Santa Sofia 78, 95123 Catania, Italy.

[§] These authors contributed equally to the paper.

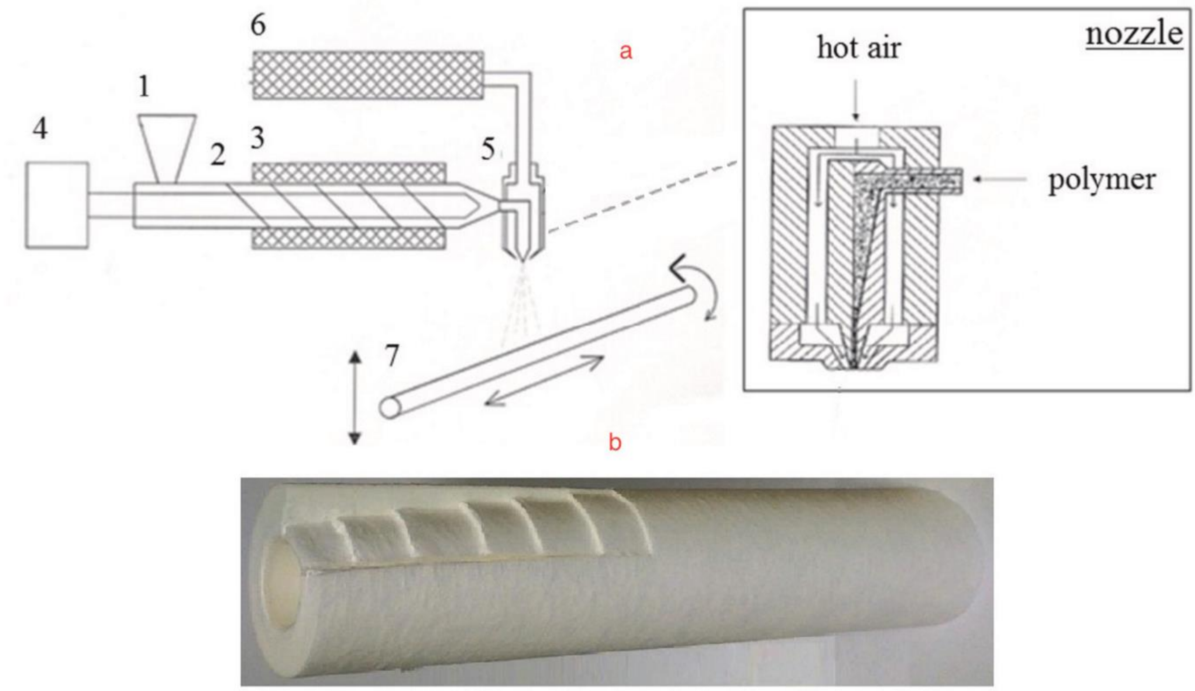

Figure S1. Melt-blown technique for production fibrous deep-bed filters

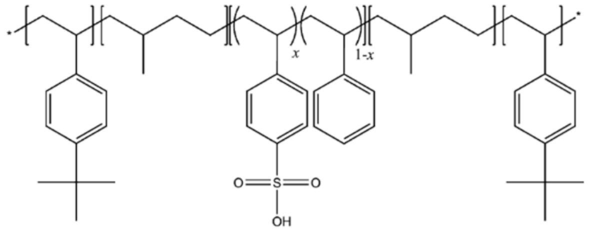

Figure S2. Molecular structure of sulfonated pentablock copolymer (s-PBC) (Nexar™).

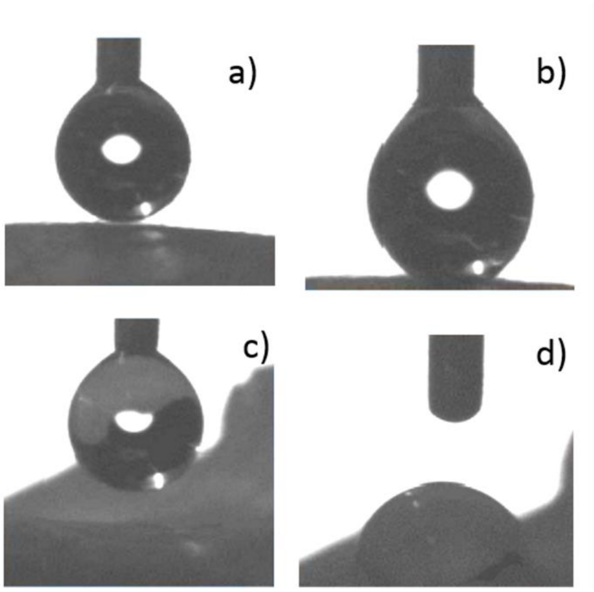

Figure S3. Optical images of contact angle measurements for PP (a,b) and s-PBC@PP (c,d) filters.

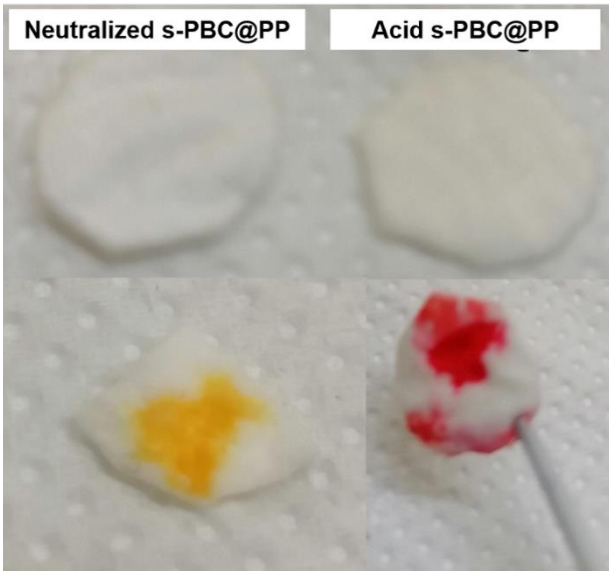

Figure S4. Photos of neutralized (on the left) and acid (on the right) s-PBC@PP coupons before (on the top) and after (on the bottom) being in contact with Methyl Orange.

[1] L. Gradon, A.; Podgorski, A.; Balazy, A., Filtration of nanoparticles in the nanofibrous filters. FILTECH EUROPE, **2005,** *5*, 178-185.
